# Heterogeneity of Early Host Response to Infection with Four Low-Pathogenic H7 Viruses with a Different Evolutionary History in the Field

**DOI:** 10.3390/v13112323

**Published:** 2021-11-21

**Authors:** Gianpiero Zamperin, Alice Bianco, Jacqueline Smith, Alessio Bortolami, Lonneke Vervelde, Alessia Schivo, Andrea Fortin, Sabrina Marciano, Valentina Panzarin, Eva Mazzetto, Adelaide Milani, Yohannes Berhane, Paul Digard, Francesco Bonfante, Isabella Monne

**Affiliations:** 1 Istituto Zooprofilattico Sperimentale delle Venezie (IZSVe), Legnaro, 35020 Padua, Italy; abianco@izsvenezie.it (A.B.); ABortolami@izsvenezie.it (A.B.); aschivo@izsvenezie.it (A.S.); afortin@izsvenezie.it (A.F.); smarciano@izsvenezie.it (S.M.); vpanzarin@izsvenezie.it (V.P.); emazzetto@izsvenezie.it (E.M.); amilani@izsvenezie.it (A.M.); FBonfante@izsvenezie.it (F.B.); imonne@izsvenezie.it (I.M.); 2 Easter Bush Campus, The University of Edinburgh, Roslin EH25 9RG, UK; jacqueline.smith@roslin.ed.ac.uk (J.S.); Lonneke.Vervelde@roslin.ed.ac.uk (L.V.); paul.digard@roslin.ed.ac.uk (P.D.); 3 National Centre for Foreign Animal Disease, Canadian Food Inspection Agency, 1015 Arlington, Winnipeg, MB R3E 3M4, Canada; yohannes.berhane@inspection.gc.ca

**Keywords:** influenza, RNA-Seq, LPAI-HPAI evolution, transcriptomic

## Abstract

Once low-pathogenic avian influenza viruses (LPAIVs) of the H5 and H7 subtypes from wild birds enter into poultry species, there is the possibility of them mutating into highly pathogenic avian influenza viruses (HPAIVs), resulting in severe epizootics with up to 100% mortality. This mutation from a LPAIV to HPAIV strain is the main cause of an AIV’s major economic impact on poultry production. Although AIVs are inextricably linked to their hosts in their evolutionary history, the contribution of host-related factors in the emergence of HPAI viruses has only been marginally explored so far. In this study, transcriptomic sequencing of tracheal tissue from chickens infected with four distinct LP H7 viruses, characterized by a different history of pathogenicity evolution in the field, was implemented. Despite the inoculation of a normalized infectious dose of viruses belonging to the same subtype (H7) and pathotype (LPAI), the use of animals of the same age, sex and species as well as the identification of a comparable viral load in the target samples, the analyses revealed a heterogeneity in the gene expression profile in response to infection with each of the H7 viruses administered.

## 1. Introduction

Influenza A viruses (AIVs) are important veterinary and human health pathogens. Avian species represent the natural hosts of all type A influenza viruses, each characterized by a specific pair of surface glycoproteins—hemagglutinin (HA) and neuraminidase (NA) [1]. To date, 16 different HA and 9 NA subtypes in most combinations have been identified from avian species [2]. Influenza A viruses infecting poultry can be divided into two distinct groups on the basis of the severity of the disease they cause. Virulent viruses cause high-pathogenicity avian influenza (HPAI) able to produce flock mortality as high as 100% in susceptible species. To date, only AI viruses belonging to the H5 and H7 subtypes have met the requirements set by the OIE Terrestrial Manual [3] to be classified as HPAI. In most cases, the remaining influenza viruses cause mucosal infections and are referred to as low-pathogenicity avian influenza (LPAI). It follows that LPAI infections with viruses of the H5 and H7 subtypes are of great concern because of their potential to evolve into the highly pathogenic form of the virus. This event has been observed to occur naturally only in these subtypes by acquiring a multibasic cleavage site (MBCS) in the HA protein. Nevertheless, the experimental insertion of an MBCS is not always sufficient to transform LPAI viruses into HPAI ones [4], meaning that virulence is also influenced by other factors [5]. However, it is still not fully understood why the H5 and H7 subtypes are more prone to evolve into highly pathogenic forms than any other AI HA subtypes [6]. Experimentally, the presence of a polybasic HA cleavage site, even in non-H5/H7 HA, can support a HP phenotype in the appropriate viral background [7,8]. In any case, all currently known HPAI outbreaks have been caused by H5 and H7 viruses. It has repeatedly been shown *in vitro*, and there is also evidence *in vivo*, that HPAI phenotypes can arise *de novo* from LPAI H5 and H7 precursor viruses [9]. In natural conditions, the species in which the switch from LPAI to HPAI had occurred was identified in only 10 out of 42 well-described HPAI primary outbreaks, with chickens being the most widely affected group [10]. Unfortunately, only in rare cases was it possible to isolate both the LPAI precursor and the respective HPAI virus from the outbreaks, which is one of the reasons that has prevented the scientific community from having a more in-depth understanding of the mechanism of pathogenicity evolution.

However, not all LPAIV H7 and H5 viruses have been found to acquire a highly pathogenic phenotype once introduced in poultry. A significant gap in knowledge of the contribution of the host in the emergence of HPAI viruses has been identified [11] as most of the studies exploring the evolution of AI pathogenicity have so far focused on viral determinants [12]. In recent years, high-throughput RNA sequencing (RNA-Seq) technology, which is a powerful way to profile the transcriptome with great efficiency and high accuracy, has been employed to investigate AIV infections [13,14,15]. RNA-Seq technology dissects the systemic changes in host gene expression in the process of infection by pathogens, which could help to uncover the dynamics and interaction mechanism between pathogens and hosts. The evolution of AIV pathogenicity is a complex event, which is the result of the interplay between various components, including the virus strain, host factors such as species, age and immune status, as well as environmental aspects such as holding type, poultry density and holding management. The studies implemented so far in humans, animal models and various *in vitro* systems clearly indicate that host innate immune response plays a critical role in viral pathogenesis and the subsequent outcome of an influenza virus infection [16,17].

In order to deepen our understanding on the host response against AIV infection of the H7 subtype, we studied the early stages of transcriptome response in the tracheal tissue of chickens infected with two pairs of H7 strains characterized by a different history of pathogenicity evolution in the field.

## 2. Materials and Methods

### 2.1. Ethics Statement

Animal experimental procedures were conducted in strict accordance with the Decree of the Ministry of Health n. 26 of 4 March 2014 on the protection of animals used for scientific purposes, implementing Directive 2010/63/EU, and approved by the Institute’s Ethics Committee (n. 18/2014).

### 2.2. Viruses

We selected two pairs of H7 low-pathogenic viruses that demonstrated a distinct ability to evolve from LPAI to HPAI under natural conditions (Figure S1, Figure S2). One pair of LP virus precursor of HP strains (precHP) included the H7N1 A/chicken/Italy/1279/1999 (Genbank accession numbers CY099594–CY099601) and the H7N3 A/chicken/BC/CN006/2004 (Genbank accession numbers OK104427-OK104434), which had respectively caused highly pathogenic outbreaks in poultry in Italy between 1999 and 2000 [18,19] and in British Columbia (Canada) in 2004 [20].

The other pair included two viruses that have not evolved into highly pathogenic strains (nevoLP), even after mid- to long-term circulation in Galliformes, namely, the H7N3 A/turkey/Italy/2962/03 (Genbank accession numbers OK104443-OK104450) and H7N2 A/chicken/Italy/1670/15 (Genbank accession numbers OK104435-OK104442) [21,22,23]. Specifically, nevoLP H7N3 circulated in poultry farms between 2002 and 2004, both in vaccinated and unvaccinated flocks. H7N2 was isolated in 2015 in Italy from a layer chicken farm. Its HA gene is phylogenetically related to other low-pathogenic H7 viruses repeatedly isolated from Italian poultry holdings until recent years (2019–2020).

### 2.3. Determination of Bird Infectious Dose 50 (BID_50_)

To standardize the infection of birds with LPAI viruses of different subtypes, we first defined the BID_50_ (bird infectious dose) of each selected strain. Our approach was based on the methods described by Swayne and Slemons [24]. Birds were challenged with serial dilutions of the selected strains. For each tested dilution, groups of 5 specific pathogen-free (SPF) white leghorn chickens (*Gallus gallus*) 4 to 6 weeks old were housed in poultry isolating units (Montair, NL). All the birds in each group were infected via the oronasal (ON) route with 100 µL of viral suspension in phosphate buffer solution (PBS) containing the corresponding EID_50_ dose (one dose per group). Tracheal swabs (FLmedical, Italy) were collected daily from Day 1 to Day 5 post infection (DPI). The samples were then processed for detection of the M gene by real-time RT-PCR [25]. The BID*_50_* was defined using the Reed and Muench method [26].

### 2.4. AIV in Vivo Phenotyping

A preliminary study was performed to evaluate tissue tropism and viral replication in different tracts of the respiratory system and the small intestine. For each virus we infected ON 12 six-week-old SPF chickens (47 days) with a dose of 500*BID_50_, to ensure infection of all the birds. At 24, 36, 48 and 72 hours post-infection (hpi), 3 birds were suppressed and choanal cleft and tracheal swabs were collected together with lung and small intestine tissues. The samples were then processed for gene M detection and quantification by real-time RT-PCR [25].

### 2.5. Chicken Infection

*In vivo* infection challenge to produce samples for RNA-Seq data analysis involved six-week-old female SPF white leghorn chickens (47 days old) infected with a dose of 500× BID_50_ to be sure that all the birds would become infected. Table 1 shows the list of viruses used for RNA-Seq. For each selected strain, 12 birds were challenged; the infection was performed via the ON route and 3 birds per time point were sacrified for the collection of organs. The RNA-Seq analysis focused on the respiratory tissue (trachea) at the early stage of infection (i.e., 24, 36, 48 and 72 hpi); we pooled together small tracheal rings collected at each location (upper, middle and lower part of the trachea). Mock infection using PBS was likewise implemented. After sampling, the target organ was preserved in RNAlater for 24 h and stored at −80 °C until processing. In addition, 3 birds per group were infected with the same dose and they were followed-up by choanal cleft and tracheal swabbing during the four timepoints to assess the viral replication load. Samples collected from these animals were processed for RNA extraction and quantitative real-time RT-PCR [25]. To monitor the composition of the viral population, an ultra-deep sequencing approach was applied to these samples, as described in Fusaro et al. (2019) [27], except that only called variants (SNPs and indels) with a frequency ≥ 1% and sequence coverage ≥ 10-fold were retained.

### 2.6. Real-Time RT-PCR

Real-time RT-PCR targeting the M gene was used to determine the BID_50_ and to assess viral replication in each experimental group, in a qualitative and quantitative set up, respectively. Swab heads were placed in 500 µL of 1X Phosphate-Buffered Saline containing antibiotics and antimycotics (PBSa) and vortexed for 30 s. Total RNA was purified from 300 µL of sample suspension using the QIAsymphony^®^ DSP Virus/Pathogen Midi Kit on a QIAsymphony^®^ SP instrument (Qiagen, Hilden, DE, Germany). Viral genome amplification was carried out using the QuantiTect Multiplex RT-PCR Kit (Qiagen, Hilden, DE, Germany), 300 nM of each primer, 100 nM of probe and 5 µL of template RNA, in a final volume of 25 µL. Each sample was tested in triplicate. Runs were performed on a CFX 96 Deep well Real-Time PCR System, C1000 Touch (Biorad, Munich, DE, Germany), under the following cycling conditions: 50 °C for 20 min, 95 °C for 15 min, followed by 40 cycles at 94 °C for 45 s, 60 °C for 45 sec. Data were analyzed with the Bio-Rad CFX Manager software (Version 3.1).

The limit of quantification of the assay (LoQ, 10^0.5^ EID_50_/100 µL) was assessed employing serial dilutions of titrated PrecHP H7N1, upon verification of identity with all the challenge viruses in the oligonucleotide hybridization regions. Ten-fold dilution series of PrecHP H7N1 were processed in triplicate along with each run to develop standard curves for virus quantification. Viral absolute quantification, expressed as EID_50_/100 µL, was performed by interpolation of the mean Ct values with the standard curve. For all the experimental groups, viral replication was plotted as the mean viral load of the three biological replicates ± SD using Prism 9.1.2 (GraphPad). For graphical purposes, specimens with a viral load below the LoQ were arbitrarily given a value of 10^0.5^ EID_50_/100 µL.

### 2.7. RNA Sequencing

RNA samples were isolated from tracheas using RNeasy Fibrous Tissue mini kit (Qiagen, Hilden, DE, Germany), following the manufacturer’s instructions. The quantity and quality of the isolated RNA samples were checked using a Qubit RNA HS assay kit (ThermoFisher Scientific, Waltham, MA, USA) and RNA 6000 Nano kit, respectively, using an Agilent 2100 Bioanalyzer (Agilent Technology, Santa Clara, CA, USA). All the samples showed the RNA integrity number (RIN) as > 8. The transcriptome library of each sample was prepared starting from 500 ng of total RNA using the Truseq Stranded mRNA library preparation kit (Illumina, San Diego, CA, USA) following the company’s protocols. Each library was run on an Agilent 2100 Bioanalyzer using an Agilent High Sensitivity DNA kit to ensure the proper range of cDNA length distribution. Sequencing was performed on an Illumina NextSeq instrument with NextSeq^®^ 500/550 High Output Kit v2 (75 cycles) in single-read [SR] mode (Illumina, San Diego, CA, USA) in order to produce about 30 million reads per sample. The raw sequencing data are available at SRA under accession number PRJNA763065.

### 2.8. Bioinformatic Analysis

Raw data were quality filtered by clipping the library adaptors and trimming low-quality ends with trimmomatic v0.38 [28]; remaining reads shorter than 50 bp were removed. High-quality data were aligned against the *Gallus gallus* reference genome (Gallus gallus 5.0, Ensembl release 94) with STAR v2.6.1c [29] and the gene count was generated using htseq-count v0.11.0 [30]. Differential expression analysis was performed with the DeSeq2 package v1.20.0 [31]. Differentially expressed genes (DEGs) were defined using FDR < 0.05 and |log2FC| ≥ 1. Gene Ontology (GO) terms were assigned to each gene by using Blast2GO v5.2.5 [32] and merged with those downloaded from Ensembl through BioMart (accessed on 5 March 2020) and from the Panther ftp site (PTHR14.0_chicken). GO enrichment was computed by Fisher’s exact test and p-values were adjusted through the Benjamini–Hochberg correction. An FDR < 0.05 was considered significant. Enrichment scores were computed as -log(FDR). Venn diagrams were produced using the online tool Venny (https://bioinfogp.cnb.csic.es/tools/venny/index.html, accessed on 14 April 2021). Pathway analysis was performed using the Ingenuity Pathway Analysis (IPA) program (Ingenuity System, QIAGEN).

## 3. Results

### 3.1. Determination of the Infectious Dose and Infection Status of Birds

The infectious doses to infect 50% of birds (BID_50_) per virus is reported in Table 1. To confirm that the dose identified for the RNA-Seq challenge was effective, in addition to the specimens used for the transcriptomic analysis, we monitored the positivity to AIV in chickens infected with the 500×BID_50_, testing choanal cleft and tracheal swabs with quantitative real-time PCR assay. In all the strains used, the replication was moderate, with slight variation amongst individuals, and no clinical signs were observed (Figure S3).

The ultradeep-sequencing analysis demonstrated that none of the positive AIV samples presented mutations or insertions of basic amino acids at the HA cleavage site (MBCS). The presence of possible virulence/adaptation markers other than the MBCS was checked for all the virus isolates used for the challenges as well as for the AIV-positive samples obtained from the infected experimental groups. The LPAI progenitor of the HPAI H7N1 Italian epidemic possessed a deletion of 22 amino acids in the NA stalk region, which is a well-known marker of viral adaptation to Galliformes, as reported in previous studies [33]. This mutation was found to be maintained in all the positive samples sequenced from the experimental group challenged with this HPAI H7N1 precursor. A deletion of 23 amino acids in the NA stalk region was identified also in the nevoLP H7N3 A/turkey/Italy/2962/03 virus and confirmed in the positive samples obtained from the respective infected experimental group. The other precHP used in our experiment, the H7N3 A/chicken/BC/CN006/2004, did not present any known virulence marker. No virulence/adaptation markers were identified in the nevoLP H7N2 A/chicken/Italy/1670/15.

### 3.2. In Vivo Phenotyping

No major differences were observed in terms of tissue tropism since all viruses revealed a preference for replication in the upper respiratory tract (Table S1). In particular, choanal cleft swabs demonstrated viral replication in all the birds at all timepoints. Lung and intestinal tract tissues were inconsistently found positive and often at later time points. NevoLP H7N2 demonstrated a shorter course of infection compared to the other viruses and viral presence was detected exclusively in the respiratory tract.

### 3.3. Global Expression Profiles

**Figure 1:**
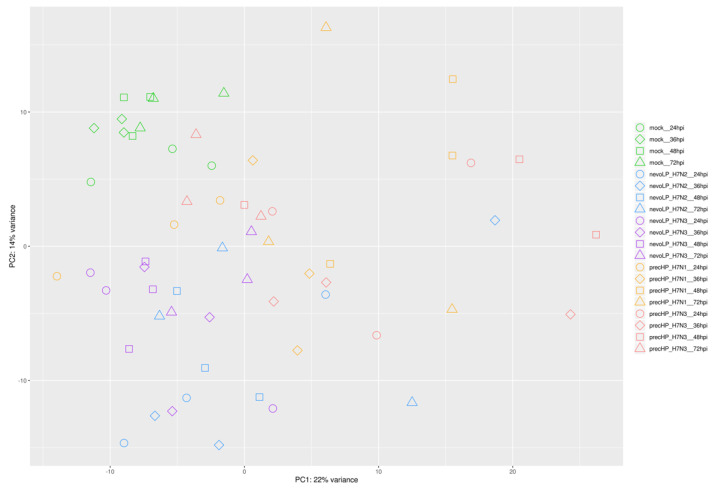
Principal component analysis of the normalized gene counts of the host genes.

RNA was extracted from the tracheas of 6-week-old SPF chickens infected with 4 different H7 strains and the gene expression was quantified using RNA-Seq technology. A principal component analysis (PCA) was used to explore the general transcriptomic profiles of the analyzed samples, limiting the analysis on the first two dimensions; i.e., the ones explaining the greatest amount of variance belonging to the data. The PCA of the normalized counts for host genes confirmed separate groupings between the mock and H7-infected tracheas (Figure 1). Both the first and second principle components contributed to the separation of the mock group from the two categories of H7 strains, accounting for one-third of the total expression variation (22% for the first component, 14% for the second).

At the single strain level, nevoLP H7N2 was well separated from nevoLP H7N3 and both showed very little distance at different timepoints. Both precursor strains showed an opposite behavior, with no clear separation between the two. A greater dispersion relating to the single timepoints was evident. No virus showed a clear separation between different timepoints, indicating the absence of a clear progression of the expression profile over time.

### 3.4. Transcriptome Analysis over Time

**Figure 2:**
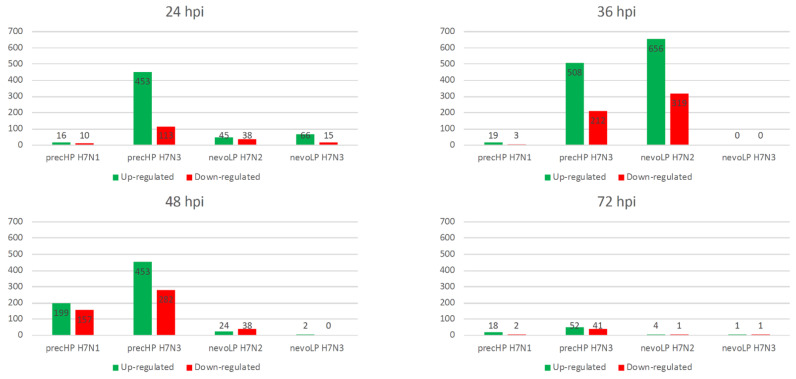
Differential expression analysis results for the four time-course H7 infections. At each timepoint, the total number of up- and downregulated genes is shown as a histogram.

We characterized the host response of four different H7 viral strains over time using four different timepoints (24, 36, 48 and 72 hpi) in trachea, a tissue known to be a very early target of viral infection. The numbers of differentially expressed genes (DEGs) show that the largest host response against each virus occurs at different timepoints (Figure 2, Table S2, Figure S4). In detail, the nevoLP H7N3 showed a mild response at 24 hpi (81 DEGs), while nevoLP H7N2 induced the highest number of DEGs (975) at 36 hpi. The precHP H7N3 induces a rapid and consistent high response over 24, 36 and 48 hpi (526, 720 and 735 DEG, respectively), after which it drops. The precHP H7N1 induces a strong response that only peaks at 48 hpi (356 DEG). It is worth noting that no DEG peak was observed at 72 hpi, except with precHP H7N3, which is the only virus that induces a mild response at this timepoint.

**Figure 3:**
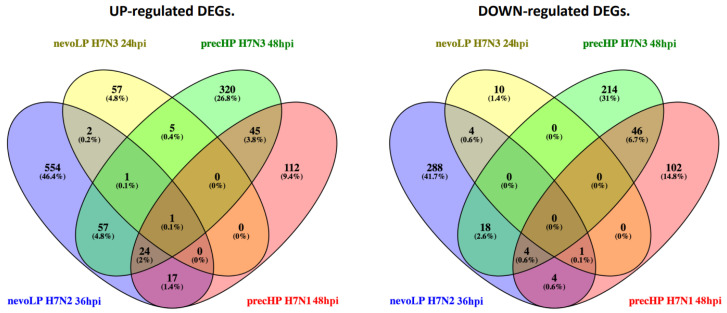
Venn diagram showing the common and specific differentially expressed genes (DEGs) between the important timepoints of different viruses. DEGs are split between up- and downregulated genes.

Focusing on the timepoints during which the host mounted the largest response, we checked how many DEGs were commonly shared among infections with different H7 strains (Figure 3). Overall, the majority of the DEGs were unique to each infection, with the nevoLP H7N2 challenge showing the highest percentage (44.3%) and the nevoLP H7N3 the lowest (3.6%). Comparing the two categories of virus, we see that the precHP strains have more common DEGs (6.6%) than the nevoLP strains (0.6%). It is worth pointing out that only one gene was commonly differentially expressed in all four infections, namely, interferon-induced protein with tetratricopeptide repeats 5 (*IFIT5*), a well-known anti-viral gene that is crucial for the innate immune response against viral infections in chickens [34,35].

### 3.5. Most Significant Differentially Expressed Genes

We identified the top differentially expressed genes (i.e., with the largest fold change) amongst all DEGs in the two pairs of H7 subtypes. Within the precursor group, the precHP H7N3 virus presents upregulated genes such as *CCAH221*, which translates chemokine AH221 [36], involved in the process of inflammation and cellular response to interferon, *IL-1* and *TNF*; *FGA*, which translates the fibrinogen alpha chain, is involved in cell apoptotic processes and exocytosis. Amongst the top downregulated genes we found *MHCIY*, coding for a major histocompatibility complex (*MHC*) class I antigen [37]; Klotho co-receptors, which encode single-pass transmembrane proteins, associated with metabolic activities and fibroblast growth [38]; *METTL11B,* encoding the methyltransferase-like *11B* [39]; *CACNG5*, translating the type II transmembrane AMPA receptor regulatory protein (neurotransmitter receptor), involved in transmission of nerve impulse [40]; and *CDH8*, translating cadherin, a cell adhesion calcium-dependent protein involved in the regulation of synapse organization [41].

The upregulated DEGs in precHP H7N1 include *S100A9*, encoding a calcium- and zinc-binding protein, which plays a prominent role in the regulation of inflammatory processes and immune response [42,43]; *ACOD1*, involved in antimicrobial response of innate immune cells and acting as a negative regulator of the Toll-like receptor (*TLR*)-mediated inflammatory innate response [44]; *SERPINB10*, translating for a protease inhibitor that may play a role in the regulation of protease activities in the cytoplasm and in the nucleus during hematopoiesis and apoptosis induced by TNF [45]; *CD72*, translated into C-type lectin domain-containing protein, playing a role in B-cell proliferation and differentiation [46]; and *CSF3R*, which encodes the receptor for colony stimulating factor 3, a cytokine that controls the production, differentiation, and function of granulocytes [47]. Amongst the top downregulated DEGs, we found *ZNF804B*, encoding a metal ion-binding protein contained in the nucleus; *GLRA3* (glycine receptor alpha 3), associated with ligand-gated ion channel, playing an important role in the downregulation of neuronal excitability and contributing to the generation of inhibitory postsynaptic currents [48]; *ANKRD1* (ankyrin repeat domain 1), induced by IL-1 and TNF-alpha stimulation, which may play an important role in endothelial cell activation [49]; and *RIMS4*, encoding for the regulating synaptic membrane exocytosis [50].

Amongst the overexpressed DEGs, both precursor strains present *IFITM1*, interferon-induced transmembrane protein 1; *IL21R*, interleukin 21 receptor; and *IFITM3*, interferon-induced transmembrane protein 3.

The nevoLP H7N2 challenge presents the following significant DEGs: *Blec2*, encoding the lectin like natural killer cell surface protein, which by similarity may be involved in natural killer (NK) cell-mediated cytolysis [51]; *CALHM3*, encoding the pore-forming subunit of a voltage-gated ion channel, associated with calcium homeostasis [52]; and *SPP2*, possibly involved in the coordination of bone turnover [53]. The top downregulated DEGs include *NKX2-3*, encoding a member of the NKX family of homeodomain transcription factors [54,55]; *Il13*, which encodes interleukin 13, an immunoregulatory produced primarily by activated Th2 cells involved in several stages of B-cell maturation and differentiation [56]; *IL1R2*, encoding a cytokine receptor that belongs to the interleukin 1 receptor family [57]; *KROX20* (zinc-finger transcription factor), a protein associated with B cell proliferation in humans; and *FOSL2* (Fos-related antigen 2), belonging to the Fos family proteins, which, together with the Jun family proteins are frequently activated by pathogens to induce cellular inflammation and immune response.

The top DEGs upregulated after nevoLP H7N3 include *CYP2AC1*, which is associated with metabolic processes; *TRIM39*, which may be involved in apoptosis and other cell cycle processes [58]; and *RHAG*, which may be engaged in ammonium transmembrane transport and erythrocyte development [59]. The most significant downregulated gene is *CRISP2*, which is thought to regulate ion channel activity [60]. Regarding the nevoLP strains, they share only one DEG, namely, *FOXE1*, encoding the forkhead box E1 protein, which acts as a thyroid transcription factor [61].

### 3.6. Gene Ontology Enrichment Analysis

**Figure 4:**
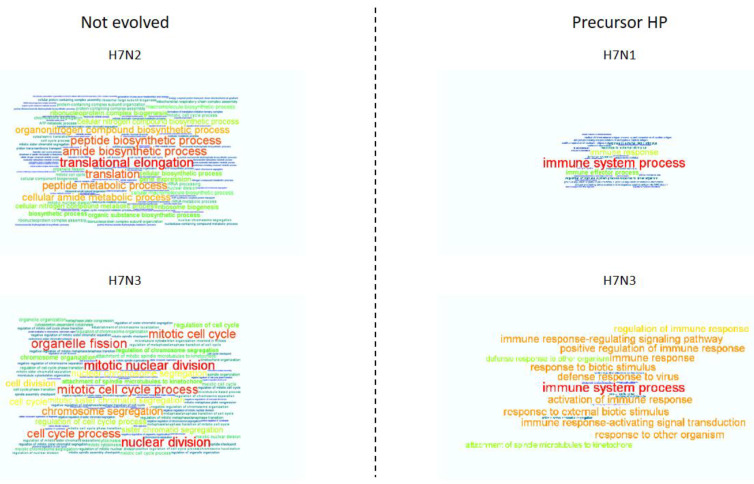
GO enrichment for the four time-course H7 infections, each with a separate word cloud. For each virus, we computed the enriched GO biological process terms at all timepoints. In case a GO term was enriched in multiple timepoints, we chose the one with the highest score, defined as −log10FDR. Font size and color (from the lowest to the highest value: blue, green, yellow, orange, red) are proportional to score. Word clouds have different values for highest score: from left to right then from top to bottom: 21.53, 7.10, 13.61 and 2.18.

To investigate the host response to AIV infection, we examined enriched biological processes in the DE gene set using Gene Ontology (GO) databases (Figure 4, Table S3). Gene Ontology analysis aims to identify those biological processes (and also cellular locations and molecular functions, if analyzed) that are impacted in the condition studied. Relationship between DEGs and GO terms is a multi:multi one, so computing enrichment on GO terms could reduce the huge list of DEGs to few biological high-level processes or, conversely, expand few DEGs into many enriched GO terms.

In general, the highest number of enriched GO terms is found at the timepoints with the highest number of DEGs: 92 enriched GO terms at 36 hpi for nevoLP H7N2, 106 at 24 hpi for nevoLP H7N3 and 31 at 48 hpi for precHP H7N1. The only exception is represented by the precHP H7N3 virus. While for the other three H7 viruses there is clearly a timepoint with a peak of differential expression, sustained high gene expression was noted at 24, 36 and 48 hpi after precHP H7N3 challenge. The number of DEGs observed at these timepoints was 566, 720 and 735, respectively, which translated into 19, 2 and 9 enriched GO terms, respectively.

Overall, enriched GO terms emerge at early timepoints for both the nevoLP H7 viruses (at 24 and 36 hpi), while for the precHP H7 viruses we found enriched GO terms at a later stage (at 48 and 72 hpi for the H7N1) or at a wider time span (from 24 to 48 hpi for the H7N3). Furthermore, for the two nevoLP H7 viruses, enriched GO terms are in greater number with higher significance when compared to those associated with the precHP H7 viruses. In fact, nevoLP H7N2 and H7N3 strains have 92 and 106 enriched GO terms, while precHP H7N1 and H7N3 viruses have 31 and 19 enriched GO terms.

The nevoLP H7N2 challenge showed 32 enriched GO terms at 24 hpi and 92 enriched GO terms at 36 hpi. Most general enriched terms at 24 hpi included chromosome segregation, cell division and cell cycle, while at 36 hpi most generally enriched terms included biosynthetic process, cellular component biogenesis, ATP metabolic process, cellular metabolic process, nitrogen compound metabolic process, primary metabolic process and organic substance metabolic process. These results suggest that at the earliest timepoint, infected cells were busy multiplying, while 12 h later they began to produce proteins, ATP and other components.

The nevoLP H7N3 virus showed 103 enriched GO terms at 24 hpi; it is interesting to note that, despite having the highest number of enriched GO terms among all the infections, it also had the lowest number of DEGs among infections (81 at 24 hpi, and only 2 at 48 and 72 hpi). The most generally enriched GO terms were partly shared with the H7N2 study, including chromosome segregation, cell division, cell cycle, microtubule-based process, cellular component organization and neural precursor cell proliferation. These results suggest a similar behavior with that of H7N2, although protein, ATP and other component production was not observed.

The precHP H7N1 challenge showed 31 enriched GO terms at 48 hpi and 2 enriched GO terms at 72 hpi. Most generally enriched terms at 48 hpi included immune response, immune effector process, response to external stimulus, response to biotic stimulus and actin filament-based process; the “response to external stimulus” term is seen also at 72 hpi. Enriched GO terms clearly indicate a host immune response as the main and only visible reaction to the viral infection.

Infection with the precHP H7N3 virus showed 19 enriched GO terms at 24 hpi, with shared ontologies seen at the following timepoints (2 at 36 hpi, both shared, 9 at 48 hpi, 56% of which are shared between such two timepoints). The most general enriched terms included immune response, response to biotic stimulus and response to external stimulus. The behavior represented by such enriched GO terms is completely overlapping with that of the precHP H7N1challenge, although to a lesser extent, given the lower number and score of enriched GO terms.

### 3.7. Pathway Analysis

**Figure 5:**
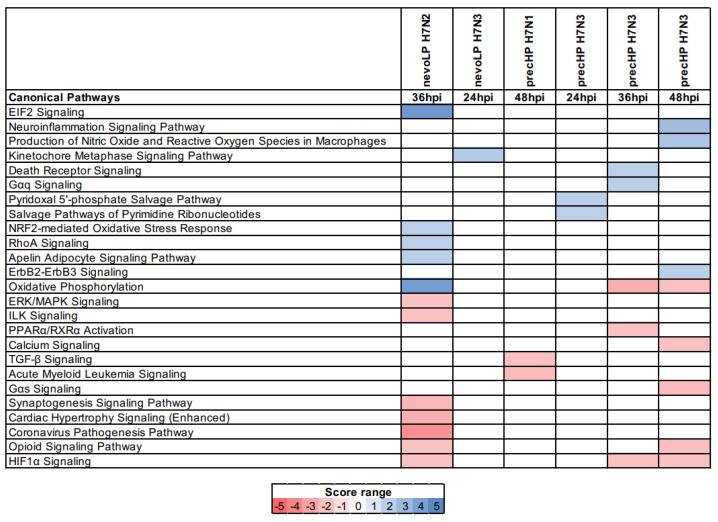
Ingenuity Pathway Analysis (IPA) results on differential expression data.

In order to define the host response to the different H7 strains more specifically, we also performed pathway analysis using Ingenuity Pathway Analysis software (IPA, QIAGEN) on DE gene sets found by differential expression analysis (Figure 5). According to the previous results based on enriched GO terms, significant activated/inhibited pathways were found at the same times where we see a peak of differential expression for each strain. Again, the precHP H7N3 challenge resulted in some significant pathways at 24 and 36 hpi, but the highest number was found at 48 hpi. A total of 25 significant pathways were identified as activated or inhibited. Overall, 88% of the pathways were specific to each strain, with only three in common between the nevoLP H7N2 and the precHP H7N3 strains; namely, “Opioid Signaling Pathway” and “HIF1alpha Signaling”, which were inhibited in both strains, and “Oxidative Phosphorylation”, which was strongly activated by nevoLP H7N2 and inhibited by precHP H7N3 (at 36 and 48 hpi).

The nevoLP H7N2 infection study showed a high number of significant pathways, accounting for 12 of them, including “Oxidative Phosphorylation” and “EIF2 Signaling” among the most activated, which are pathways tied to protein production and metabolism activation. The nevoLP H7N3 virus showed only one significant pathway, “Kinetochore Metaphase Signaling Pathway”, that was activated. This pathway is involved in mitotic events and reflects perfectly the GO enrichment results.

The precHP H7N1 challenge showed two inhibited pathways, “TGF-beta Signaling” and “Acute Myeloid Leukemia Signaling”. The latter pathway is linked to hematopoietic differentiation, signal transduction and, in general, to cellular growth, development and proliferation. TGF-beta Signaling involves a family of structurally related cytokines that induces a multitude of effects, including controlling the proliferation, differentiation, migration and apoptosis of many different cell types. Lastly, the precHP H7N3 viral infection resulted in 13 significant pathways overall, partly shared across different timepoints (2 at 24 hpi, 5 at 36 hpi and 8 at 48 hpi), with the “Neuroinflammation Signaling Pathway” and “Production of Nitric Oxide and Reactive Oxygen Species in Macrophages” as the most activated.

In general, pathway analysis seems only partially in agreement with the GO enrichment results. More specifically, for viruses belonging to the nevoLP category, the significant pathways refer to general metabolic functions such as protein synthesis, ATP production (for nevoLP H7N2) and cell division (for nevoLP H7N3). In the precHP H7N3 challenge, a clear activation of the immune system is observed according to the IPA results, while for the other virus of the same pair, precHP H7N1, it is not obvious to assess the exact biological meaning of the significantly inhibited pathways observed.

## 4. Discussion

Once an LPAIV of the H7 or H5 subtype affects poultry, it has the possibility of mutating to HPAIV, resulting in a severe epizootic with potentially up to 100% mortality. This mutation from LPAIV to HPAIV has a catastrophic economic impact on poultry production [62] and could also have implications for human health, as HPAI viruses do occasionally infect people (https://www.who.int/news-room/fact-sheets/detail/influenza-(avian-and-other-zoonotic; accessed on 10 June 2021). Despite the impact of HPAI viruses and their relevance to animal and human health, the selective factors driving the emergence of HPAI viruses from LPAI ones have remained elusive [63], thus reducing the possibility of improving early warning and response. So far, research studies have analyzed this key topic from a viral perspective. The HA subtype is known to be the most relevant viral factor to switch from a LPAI precursor to a HPAI phenotype as, to date, only the H5 and H7 subtypes seem to be prone to the incorporation of multiple basic amino acids in the HA0 cleavage site. However, the reasons why only the H5 and the H7 subtypes may acquire an HPAI phenotype are not yet fully understood, as it remains unclear why not all H5 and H7 viruses systematically evolve into HP [64,65]. There is experimental evidence demonstrating that the insertion of a multiple basic HA cleavage site, even in non-H5/H7 HA, can support a highly pathogenic phenotype in the appropriate viral background [7,8]. Our study has pioneered a new approach to the investigation of the factors that possibly drive the evolution of pathogenicity in avian influenza viruses, as it has enabled a switch from virus-focused methods for investigating the mechanisms of HPAI emergence to an unbiased, discovery-based concept through the application of RNA-Seq of host transcriptomes. We analyzed and compared the early host reactions to infection with four viruses, all belonging to the H7 subtype with different evolutionary histories in the field. More specifically, we used as the challenge viruses two LPAI viruses that mutated into HPAI at the early stages of two distinct epidemics, namely, the Canadian 2004 H7N3 and the Italian 1999–2001 H7N1 outbreaks, and two LPAI viruses not related to any HPAI emergence despite prolonged circulation in domestic poultry. Chicken (*Gallus gallus*) was selected as the candidate host for the in vivo studies as it represents the most common host species in which the transition from a low- to high-pathogenic form has occurred in the past [10].

Despite the inoculation of a normalized infectious dose of viruses belonging to the same subtype (H7) and pathotype (LPAI), the use of animals of the same age, sex and species as well as the identification of a comparable viral load in the target samples, the analyses and comparisons of the transcriptome data highlighted the uniqueness of the response among the four experimental groups. This evidence is in line with the results of previous comparative studies implemented using viruses with different virulence in *in vitro* and in animal models, which demonstrated that differences in the host response cannot be attributed solely to differences in viral replication but rather more to the properties of the individual influenza A virus [66,67,68,69,70,71]. In our study, we aimed to use viruses that have demonstrated very different characteristics in terms of phenotype evolution but in which no genomic differences crucial to LP–HP transition have been recognized. This choice may constitute an additional element of complexity for the interpretation of differentially expressed genes or identified cellular pathways peculiar to each virus. For all these reasons, the further recognition of common elements shared within the host response to the same set of viruses becomes particularly challenging.

Furthermore, the timing of viral infection [72] is another factor that may differ among viruses even when using the normalized infectious doses. Monitoring the course of infection over time using choanal cleft and tracheal swabs showed that chickens were infected in all the chosen timepoints without a clear peak of replication. This observation was further strengthened by our differential expression analysis, which showed that each virus triggered a host response at a particular timepoint, which did not correlate with the viral loads found on the analyzed swabs. Analysis of choanal cleft and tracheal swabs also highlighted a high variability among biological replicates [73,74], indicating that single birds can mount a distinctive response to infection, despite sharing the same genetics, age and sex. This is probably due to the high complexity of a biological system such as an animal model in comparison to less complex *in vitro* models, such as cell cultures or tissue explants, which mainly have been used in previous RNA-Seq studies. Similar studies performed in complex whole organisms should take into consideration the need to increase the number of animals used as biological replicates, to the benefit of greater accuracy and reliability of the data obtained. This raises an ethical question of great importance, considering the need of compliance with the 3Rs principle (reduction, refinement and replacement).

Nevertheless, our analyses revealed the existence of common biological functions induced by non-evolved LPAI strains compared to LPAI precursors of HPAI viruses. In particular, enrichment analysis of differentially expressed genes showed that genes involved in immune response were well represented in chicken tracheas infected by both HPAI precursors, while genes involved in metabolic processes, protein production and cellular division were more represented in tissues from birds infected with non-evolved strains. The greater variability observed in birds infected with nevoLP strains may be explained by the fact that viruses with this evolutionary behavior (not evolved) naturally outnumber those responsible for LP–HP transition. Considering that only a limited number of circulating viruses experience the switch from low to high pathogenicity in the field, based on the data generated here, we could speculate that the host response induced by these strains presents less variability. Enriched pathways partially confirmed this hypothesis, showing the activation of pathways, such as the production of nitric oxide and ROS (reactive oxygen species), in macrophages in the experimental group challenged with the precHP H7N3. This pathway represents a central process to the control of viral infections by the innate immune system [75]. On the other hand, within the nevoLP group, pathways clearly related to metabolic activities, such as oxidative phosphorylation, which is crucial to produce adenosine triphosphate (ATP), were identified in the H7N2 challenge. At the single gene level, it is interesting to note that one of the most upregulated genes after challenge by precHP strains was *CSF3R*, whose encoded protein is a member of the family of cytokine receptors and may also function in some cell surface adhesion or recognition processes. This receptor is essential for granulocytic maturation and plays a crucial role in the proliferation, differentiation and survival of cells along the neutrophilic lineage. In addition, it may function in some adhesion or recognition events at the cell surface. Despite the results of the enrichment analysis, components involved in the innate immune response were also expressed differentially at the single gene level for non-evolved strains. More specifically, *IFITM1*—interferon-induced transmembrane protein 1, *IL21R*—interleukin 21 receptor and *IFITM3*—the interferon-induced transmembrane protein 3 (IFITM3), were overexpressed, as observed after infection with precHP viruses. These identified upregulated genes encode for proteins involved in immune response to viral infection; in particular, the IFN-induced antiviral proteins (IFITM1 and IFITM3) inhibit the entry of viruses to the host cell cytoplasm, allowing endocytosis but at the same time preventing subsequent viral fusion and release of viral contents into the cytosol. IL21 is important for the proliferation and differentiation of T cells, B cells and natural killer (NK) and dendritic cells. The ligand binding to this receptor leads to the activation of multiple downstream signaling molecules, including *JAK1*, *JAK3*, *STAT1* and *STAT3*. Knockout studies of a similar gene in mice suggest a role for this gene in regulating immunoglobulin production [76]. Additionally, the group infected with the nevoLP H7N2 virus presents downregulated genes belonging to the immune response: *IL1R2*, which encodes a cytokine receptor that belongs to the interleukin 1 receptor family, binding interleukin alpha (IL1A) and interleukin beta (IL1B); and the interleukin 1 receptor, type I (*IL1R1/IL1RA*), acting as a decoy receptor that inhibits the activity of its ligands. While it is expected to find regulation of immune genes in experimentally infected chickens and it is a clear evidence of an active infection, enrichment analysis highlights the fact that the first line of defense against IAVs is initiated with a different magnitude in those viruses that switched from an LP to a HP genotype and phenotype in the field.

It is also worth noting that for the precursor strains, the analysis of DEGs found the presence of *CACNG5, GLRA3* and *RIMS4* genes associated with synaptic transmission, which can be interpreted as an interesting finding in light of the neurotropism often associated with highly pathogenic AIVs. In addition, both for the commonly expressed genes and the enriched GO biological process, the precHP H7N1 and H7N3 challenges presented evidence of the activation of immunological and inflammatory response (*IFITM1*, *IFITM3*, *IL21R*). The only differentially expressed gene shared by the non-evolved low-pathogenic strains is *FOXE1*. Considering both its uniqueness and particular function (transcription factor involved in thyroid morphogenesis), its role and function in the context of influenza infection will need to be further investigated in future studies. Interestingly, the nevoLP H7N3-infected group presented a notably lower number of DEGs compared to the other viruses, despite a comparable replication rate assessed with tracheal swabbing. This is the only strain used in the study isolated from turkey (*Meleagris gallopavo*) and not chicken (*Gallus gallus*). An incomplete adaptation to the host may explain why the number of DEGs found for this virus is notably lower compared to the amount found in the other infections, despite having a similar number of enriched GO terms

Our challenge study demonstrated that the number and type of DEGs can profoundly vary between viruses and even within the same HA subtype, species and tissue. These results provide an additional reference to explain the great diversity observed in the pathobiological behavior of each avian influenza strain and the consequent difficulties in predicting evolutionary trajectories of any emerging AIV. Although our investigation has not identified a specific set of DEGs to be used as predictors of virus pathogenicity evolution, it is interesting to highlight that the analysis of the biological significance of the alterations in gene expression revealed some commonalities. In particular, GO enrichment analysis demonstrated that nevoLP H7N2 and nevoLP H7N3 viruses strongly impacted on the cell machinery, resulting in the activation of metabolic processes, protein production and cellular division, which are all crucial components to produce the next generation of viruses. As expected, after a virus challenge in a susceptible host, all the viruses induced a different expression of genes involved in the innate immune response. However, the magnitude of this response was different in the four experimental groups. More specifically, it is worth noting that both the precHP viruses showed a higher number of general enriched terms related to immune response. Host immunity is a well-known driver of AI evolution [77]. Thus, it cannot be excluded that an exacerbated innate immune response may activate a cascade of events aiming at the resolution of the infection, which can force a virus possessing predisposition to acquire a polybasic HA cleavage site [6] to activate multiple strategies to facilitate infection. Indeed, the acquisition of MBCS enables the virus to replicate in multiple tissues and, as it generally happens with emerging mutations that provide an enhanced replication ability, this is an advantageous trait at the viral population level [78]. Despite the limitations posed by the small number of naturally occurring H7 HPAI precursors available, the data generated here encourage the implementation of additional transcriptome studies that may eventually explore the impact of host response in shaping AIV evolution. In this respect, the progress in third-generation sequencing technologies will facilitate dual RNA-Seq of a pathogen and host at the single-cell level, increasing our chance to understand how AIVs hijack certain cellular pathways for their benefit, while interfering with others to evade host defense mechanisms.

## Figures and Tables

**Table 1:** Viruses used and infectious dose identified.

Subtype	Virus	Type	BID_50_
H7N1	A/chicken/Italy/1279/1999	Precursor HP (precHP)	10^4.5
H7N3	A/chicken/BC/CN006/2004	Precursor HP (precHP)	10^3.83
H7N2	A/chicken/Italy/1670/15	LP not evolved (nevoLP)	10^4.5
H7N3	A/turkey/Italy/2962/03	LP not evolved (nevoLP)	10^3.17

## Data Availability

The complete genomes used in this study are available from GenBank under the following accession numbers: CY099594-CY099601 (precHP H7N1 A/chicken/Italy/1279/1999), OK104427-OK104434 (precHP H7N3 A/chicken/BC/CN006/2004), OK104435-OK104442 (nevoLP H7N2 A/chicken/Italy/1670/15) and OK104443-OK104450 (nevoLP H7N3 A/turkey/Italy/2962/03). The sequencing data generated in this study is available from SRA under accession number PRJNA763065.

## Supplementary Materials

The following are available online at https://www.mdpi.com/article/10.3390/v13112323/s1, Figure S1: Phylogenetic tree of not evolved H7 strains and precursor H7N1; Figure S2: Phylogenetic tree of precursor H7N3; Figure S3: Viral replication in choanal cleft and tracheal swabs by M-gene real-time RT-PCR. For all the experimental groups, values are expressed as the mean viral load (EID50/µL) of the three biological replicates ± SD, over the observation period. The dotted line corresponds to the limit of quantification of the assay; Figure S4: Volcano plot of genes that resulted as differentially expressed in at least one viral challenge at its peak timepoint; Table S1: For each virus, 3 animals were tested and sacrified at each timepoint. We reported total number of positive animals over 3 and, if any, average Ct values (in parenthesis); Table S2: Log2FC e FDR values found for each comparison done in differential expression analysis; Table S3: Significant enriched GO terms and their scores found for each comparison done in differential expression analysis.
