# Supplementary material for: Heterogeneity of Early Host Response to Infection with Four Low-Pathogenic H7 Viruses with a Different Evolutionary History in the Field
Source: Viruses. 2021 Nov 21;13(11):2323. doi: 10.3390/v13112323 (PMC8620788; doi:10.3390/v13112323)
Supplement: Supplementary file 1 [file viruses-13-02323-s001.zip › Supplementary_Material/Supplementary Figure S3.pdf]

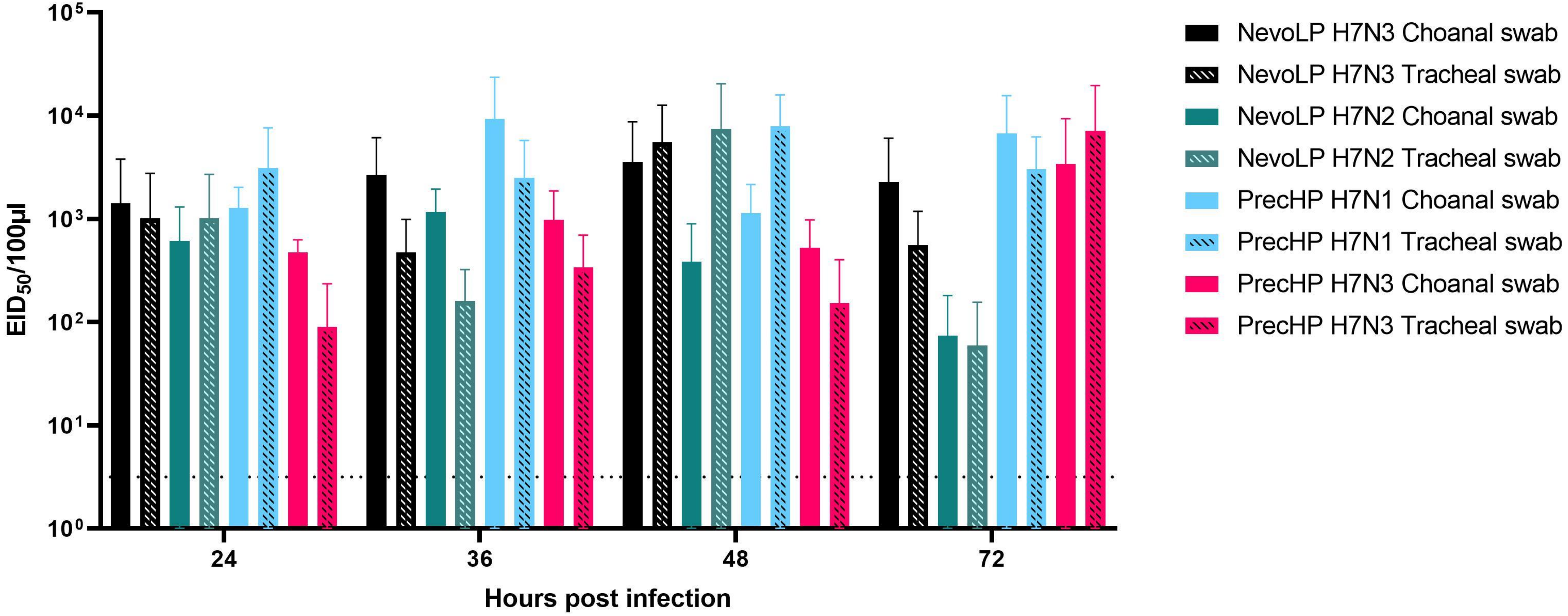

Supplementary Figure3: viral replication in choanal cleft and tracheal swabs by M-gene real-time RT-PCR. For all the experimental groups, values are expressed as the mean viral load (EID_{50}/µl) of the three biological replicates ± SD, over the observation period. The dotted line corresponds to the limit of quantification of the assay.
