# Supplementary material for: Heterogeneity of Early Host Response to Infection with Four Low-Pathogenic H7 Viruses with a Different Evolutionary History in the Field
Source: Viruses. 2021 Nov 21;13(11):2323. doi: 10.3390/v13112323 (PMC8620788; doi:10.3390/v13112323)
Supplement: Supplementary file 1 [file viruses-13-02323-s001.zip › Supplementary_Material/Supplementary Figure S4.pdf]

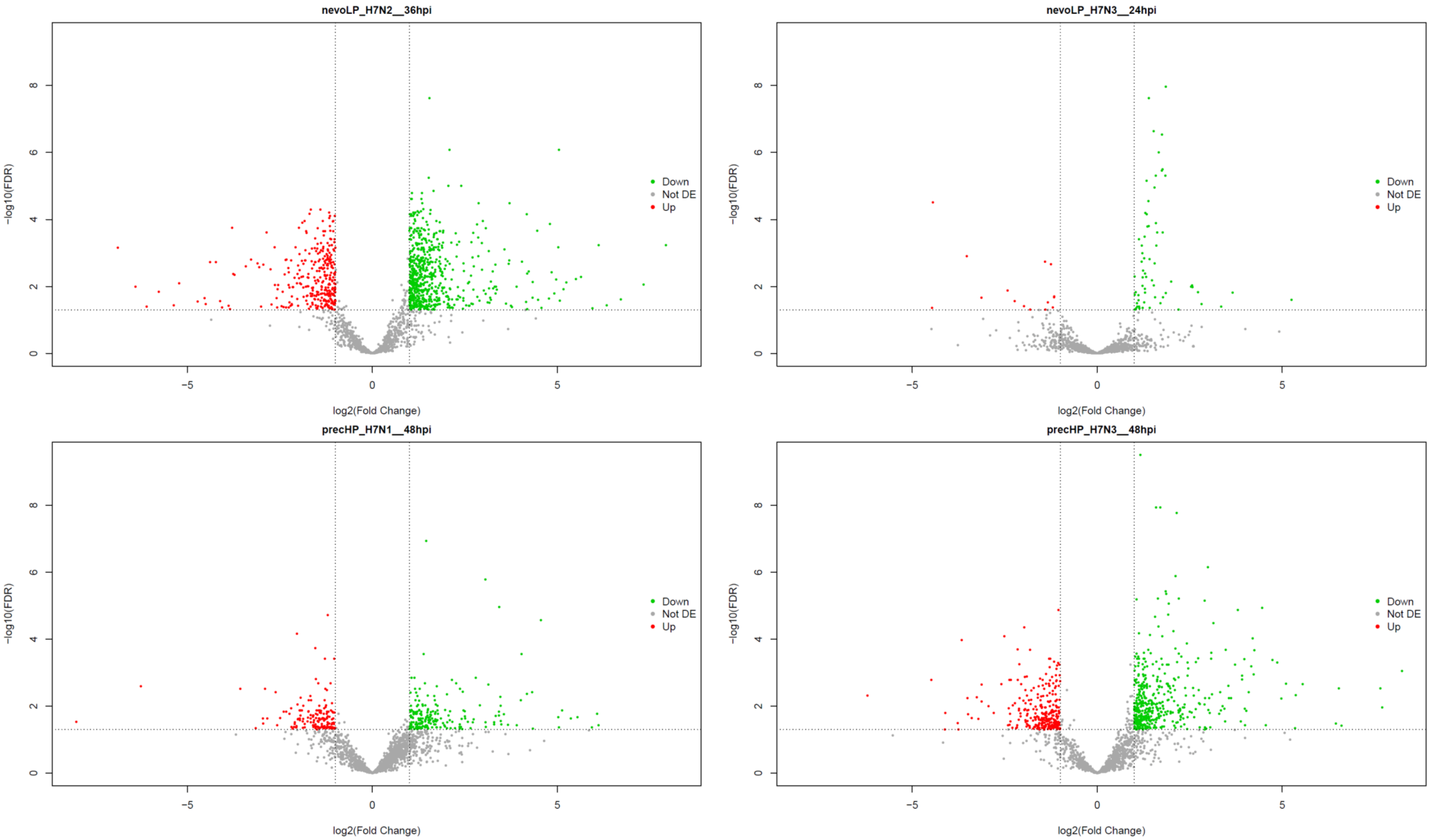

Supplementary Figure 4: volcano plot of genes that resulted as differentially expressed in at least one viral challenge at its peak timepoint.
