# Supplementary figures and images for: Heterogeneity of Early Host Response to Infection with Four Low-Pathogenic H7 Viruses with a Different Evolutionary History in the Field
Source: Viruses. 2021 Nov 21;13(11):2323. doi: 10.3390/v13112323 (PMC8620788; doi:10.3390/v13112323)

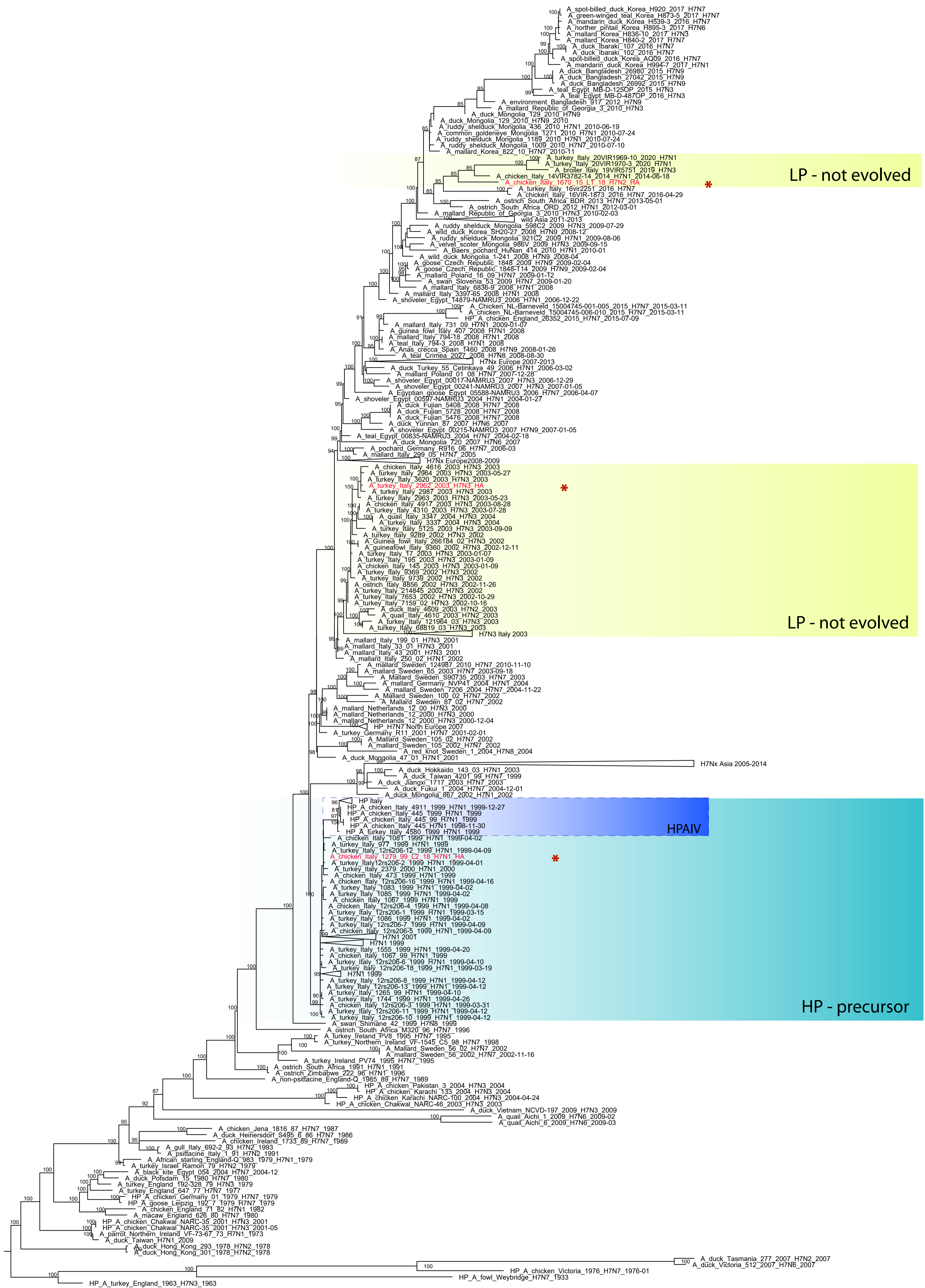

Supplement: Supplementary file 1 [file viruses-13-02323-s001.zip › Supplementary_Material/Supplementary Figure S1.pdf]

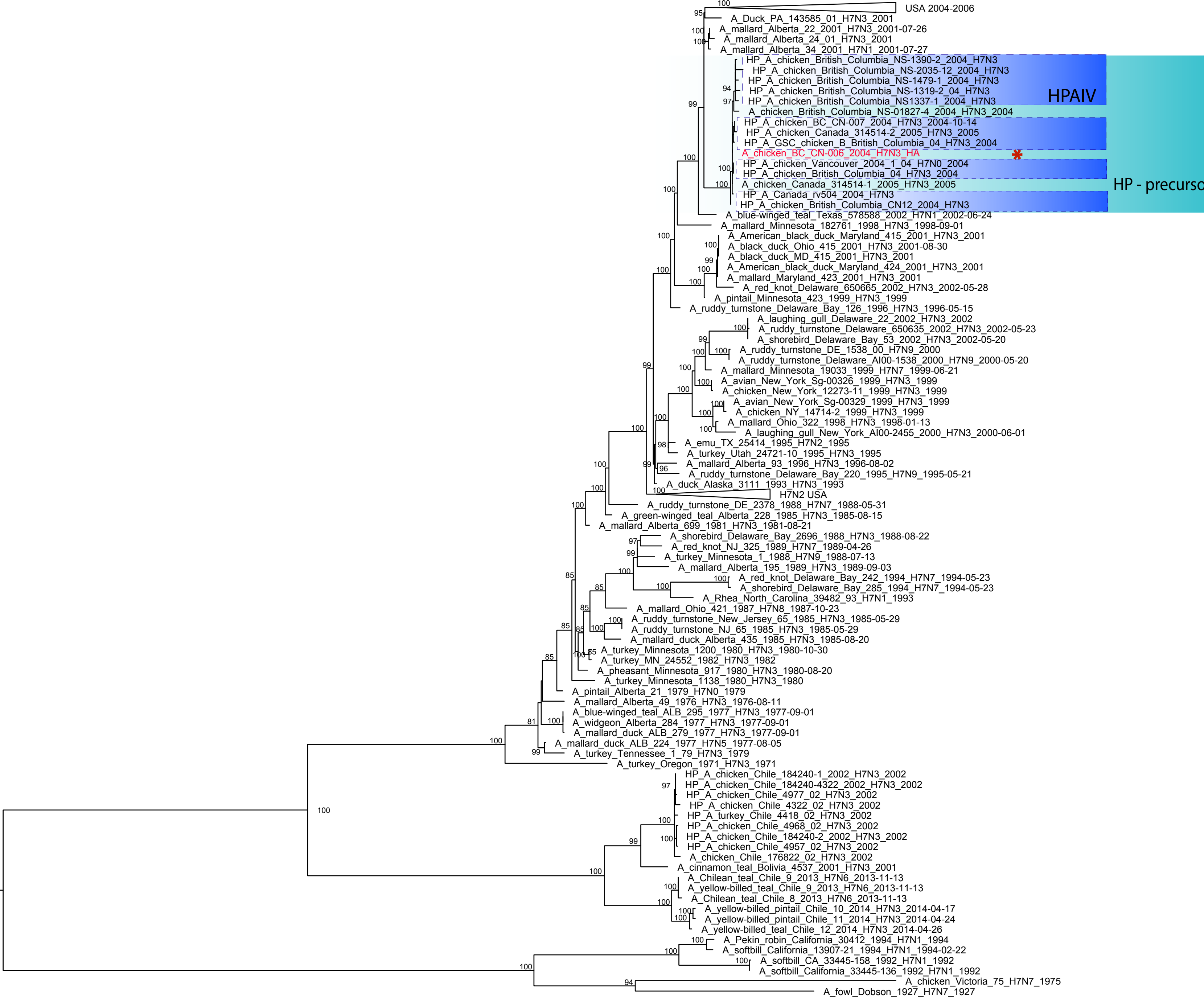

0.05

Supplement: Supplementary file 1 [file viruses-13-02323-s001.zip › Supplementary_Material/Supplementary Figure S2.pdf]
